# Fetal heart rate patterns associated with acidemia at fetal blood sampling during labor—An observational study

**DOI:** 10.1111/aogs.70347

**Published:** 2026-08-31

**Authors:** Erika Gyllencreutz, Klara Gröndal, Desiré Malm, Kari Johansson, Malin Holzmann

**Affiliations:** ^1^ Department of Women's and Children's Health Karolinska Institutet Stockholm Sweden; ^2^ Department of Obstetrics and Gynecology Östersund Hospital Östersund Sweden; ^3^ S:t Göran Hospital Stockholm Sweden; ^4^ Department of Medicine Solna Karolinska Institutet Stockholm Sweden; ^5^ Medical Unit Pregnancy and Delivery Care Karolinska University Hospital Stockholm Sweden

**Keywords:** cardiotocography, fetal acidemia, fetal blood sampling, fetal heart rate monitoring, fetal hypoxia, fetal surveillance

## Abstract

**Introduction:**

Cardiotocography (CTG) is used worldwide for fetal surveillance with the aim to detect fetal hypoxia and aid timely intervention. The method is associated with shortcomings; for example, low interobserver reliability and low specificity for fetal hypoxia. Fetal scalp blood sampling (FBS) is an adjunct method to improve the specificity for fetal acidemia. Given the updated Swedish CTG classification introduced in 2017, the aim of this study was to evaluate which fetal heart rate (FHR) patterns are most strongly associated with acidemia as determined by FBS.

**Material and Methods:**

An observational cohort study on all fetuses that during the year 2019 underwent indicated FBS during labor. The data were collected from the Stockholm‐Gotland Perinatal cohort. CTG traces were retrospectively interpreted, focusing on the last hour prior to FBS. All analyses were made with the bedside meter Lactate Pro 2™, and a lactate concentration > 7.3 mmol/L was used as a proxy for fetal acidemia.

**Results:**

During the study period, 1116 fetuses underwent FBS (8%–9% of the laboring population). Of the 1116, 148 (13%) had acidemia at the first FBS, and 38% had more than one FBS. The corresponding number of fetuses with acidemia at the last FBS was 239/1116 (21%). The FHR patterns with the strongest association with acidemia at FBS were fetal tachycardia and/or reduced variability with repetitive late decelerations (20% at 1st FBS, 34% at last FBS) or severe variable decelerations (22% at first FBS, 33% at last FBS). At the last FBS, 6/23 (26%) fetuses with a normal baseline frequency, normal variability, and non‐repetitive decelerations since >60 min of the types severe variable or late had acidemia. These FHR patterns would be classified as normal with the current classification.

**Conclusions:**

Among high‐risk fetuses with FHR patterns giving rise to FBS, the FHR patterns most often associated with fetal acidemia during labor are tachycardia and/or reduced variability combined with repetitive late or severe variable decelerations. All FHR patterns with the highest risk of acidemia are classified as abnormal in the current Swedish CTG classification system. However, longstanding milder FHR alterations warrant increased attention.

## Graphical abstract

The Swedish fetal heart rate (FHR) classification from 2017 identifies most FHR patterns associated with fetal acidemia as non‐normal, according to fetal blood sampling (FBS) in immediate connection to the ongoing FHR pattern. However, long‐standing FHR patterns with non‐repetitive decelerations of the types severe variable and late warrant increased attention.

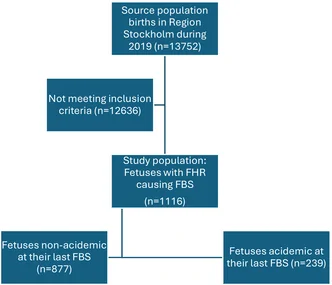

**Abbreviations**
Bpm: beats per minute
CTG: cardiotocography
FBS: fetal blood sampling
FHR: fetal heart rate
FIGO: the International Federation of Gynecology and Obstetrics

**Key message**

The Swedish fetal heart rate classification from 2017 identifies most patterns associated with fetal acidemia as non‐normal. However, longstanding milder fetal heart rate alterations warrant increased attention.

## 1 INTRODUCTION

The aim of intrapartum fetal surveillance is to prevent severe fetal metabolic acidosis and ultimately neonatal encephalopathy, and perinatal death related to labor. Cardiotocography (CTG) is the main method used for fetal surveillance in developed countries.^1^ CTG has a high sensitivity, but low specificity to identify fetuses with hypoxia, which means a high proportion of false positive tests, increasing the risk of interventions without benefit for the fetus.^1^ Unnecessary operative interventions during labor may cause maternal and neonatal harm, whereas delayed interventions can cause harm to the neonate.^1^, ^2^

Fetal scalp blood sampling (FBS) during labor is a supplementary tool to CTG that can be added when the fetal heart rate (FHR) is interpreted as non‐reassuring or abnormal but not indicating immediate need for delivery. FBS aims to detect fetal acidemia and assist in guidance of timely intervention, such as cesarean delivery or vacuum extraction delivery, if needed.^3^, ^4^ FBS was first introduced in the 1960s and initially the level of pH in the fetal blood was tested.^5^ During the 1980s, analysis of lactate concentration in FBS was developed.^6^ Analysis of pH requires a relatively large amount of fetal blood (30–50 mikroL) and does not discriminate between respiratory and metabolic acidosis. Lactate analysis is performed bedside and requires a smaller amount of blood (0.5–5 mikroL),^7^ which entails shorter time from sampling to test result, and significantly lower failure rate.^8^, ^9^ When anaerobic metabolism occurs, lactic acid is produced as an end product. The lactic acid molecule splits within seconds into negative lactate ions and positive hydrogen ions which make the lactate concentration a proper proxy for hydrogen ion concentration (i.e., pH) specifically due to lack of oxygen and not confounded by respiratory acidocis, that is, when the hydrogen ion concentration increases due to accumulation of carbon dioxide but not hypoxia.

In 2015, the International Federation of Gynecology and Obstetrics (FIGO), as the first update since 1987, published a new guideline for CTG classification.^10^ Based on the 2015 FIGO classification, Sweden revised its CTG classification, which was introduced in 2017.^11^ More FHR patterns are normalized with the revised Swedish classification, with the aim to increase CTG specificity without jeopardizing sensitivity for fetal hypoxia. Some definitions are identical to FIGO 2015, but not all.

In the revised Swedish classification, the cutoff for tachycardia is increased to 160 beats per minute (bpm), compared to 150 bpm in the previous classification. Accelerations are no longer mandatory for a normal intrapartum CTG. Decelerations need to be repetitive (occur with ≥50% of the contractions) to be considered abnormal, except for one prolonged deceleration (>5 min). Repetitive early and simple variable decelerations (i.e., duration of less than 1 min) are considered normal. Severe variable decelerations, with a duration of 1 min or more, are classified as non‐reassuring if the baseline FHR and variability are normal. However, with the addition of tachycardia and/or reduced variability the CTG is classified as pathological.^12^, ^13^

The revised Swedish classification has been criticized as being too liberal, with a decreased sensitivity as a result, compared to the previous Swedish classification.^14^, ^15^, ^16^ In two retrospective studies of CTG traces, Ekengård et al. concluded that the sensitivity of pathological FHR patterns to identify acidemia defined by umbilical cord blood pH, had decreased from 95% to 77% with the new classification, whereas the specificity had increased from 90% to 97%.^14^, ^15^ A retrospective study from the Swedish Pregnancy register, without access to information on FHR patterns, showed that a higher proportion of newborns had acidosis in umbilical artery and/or low Apgar scores during 2 years after the implementation of the revised Swedish CTG classification compared to 2 years with the previous classification.^13^ None of those studies can however be said to entail strong evidence of causation.

A previous study investigated how different FHR patterns were associated with increased lactate concentration at FBS. The study concluded that fetal tachycardia in combination with late and/or severe variable decelerations had the highest correlation with fetal acidemia among sampled fetuses^17^ The study was performed in the Swedish context, but during the years 2009–2011, when the previous CTG classification was in use, and hence, with broader recommendations for FBS.

Considering the revised Swedish CTG classification, with different indications for FBS compared to the previous, we believed it would be of interest with a new study on the relations between different FHR patterns and fetal acidemia in the Swedish context. The aim of our study was to identify which FHR patterns during labor were considered indications for FBS in the real‐world setting, and moreover the proportions of FHR patterns associated with fetal acidemia at FBS following the implementation of the revised CTG classification in Sweden.

## 2 MATERIAL AND METHODS

The study is an observational cohort study. The data was collected from the Stockholm‐Gotland Perinatal cohort, a research registry with detailed prospectively collected information from medical records antenatally, intrapartum and postpartum of the person giving birth and of the neonate in the region of Stockholm‐Gotland.^18^ The data was collected from the entire year of 2019.

Inclusion criteria were gestational age > 34 weeks, singleton pregnancy, and cephalic presentation. FBS had been performed during the delivery due to an abnormal FHR pattern according to the doctor on call. The study period, and thereby the anticipated sample size, was determined based on the power analysis used for a previous study of a similar cohort.^17^

Intrapartum CTG surveillance was conducted according to Swedish guidelines^19^ and interpreted in accordance with the revised Swedish CTG classification which was introduced in 2017.^11^ The CTG trace was interpreted by the doctor on call as normal, non‐reassuring or pathological. If difficult to assess, or if interpreted as non‐reassuring or pathological, the doctor on call could decide on performing FBS.

FBS was performed according to clinical routine: approximately 0.5 microL of fetal scalp blood was collected after wiping the fetal scalp dry from amniotic fluid. The blood was analyzed bedside with the lactate meter Lactate Pro 2™ at all units. Lactate concentrations >7.3 mmol/L were defined as fetal acidemia and constituted the main outcome.^20^, ^21^

One of the authors, a senior obstetrician with clinical and research experience within the field of FHR monitoring (MH), interpreted all CTG traces in retrospect, focusing on the last 60 min prior to each FBS. The observer was blinded regarding the lactate concentrations at FBS as well as the clinical information. The observer assessed the CTG and determined: baseline FHR (continuous variable rounded to closest 5 bpm), variability (absent [<2 bpm], decreased [2–4 bpm], normal [5–25 bpm], increased [>25 bpm]), accelerations (present or not), decelerations (none, early, late, simple variable [duration < 1 min], severe variable [duration ≥ 1 min], prolonged, combined, not assessable), whether decelerations were repetitive (occurring with ≥50% of contractions) or non‐repetitive (occurring with <50% of contractions), uterine contractions (number per 10 min), the duration of the specific FHR pattern prior to FBS (<20 min, 20–40 min, 41–60 min, >60 min). The CTG traces were at a later point categorized into 14 different groups based on different combinations of baseline FHR, variability, type and frequency of decelerations.

In labors with multiple (more than one) FBS, the CTG traces prior to the first and the last FBS were interpreted. Hence, in labors with one FBS, this sample is presented both as the first and as the last lactate. If there were repeated samples within a 5‐min period, the result with the lowest lactate concentration was chosen, since contamination with amniotic fluid can explain higher readings.^22^

### 2.1 Statistics

Statistical Package for Social Sciences software (IBM® SPSS® version 25 for Windows; IBM Corp.) was used for descriptive analyses. Continuous variables are presented as means with standard deviations and medians with interquartile ranges (25th–75th centiles). Categorical variables are presented as numbers and percentages. The sample size was estimated based on a previous power analysis for a similar cohort study.^17^ Due to the revised CTG classification, the reference group was smaller than anticipated. Given the sparse data in several groups, proportions of outcomes were reported with 95% confidence intervals (CIs) calculated using Wilson's score method,^23^ and absolute risk differences with Newcombe's method 10.^24^ *p*‐values were computed using Fisher's exact test.^25^ For each of the first‐ and last‐FBS analyses, 13 groupwise comparisons with the respective reference group were performed. Within each analysis, Bonferroni adjustment was applied by multiplying each unadjusted *p*‐value by 13. Adjusted p‐values exceeding 1.00 were capped at 1.00. A Bonferroni‐adjusted *p*‐value < 0.05 was considered statistically significant.

## 3 RESULTS

**FIGURE 1:**
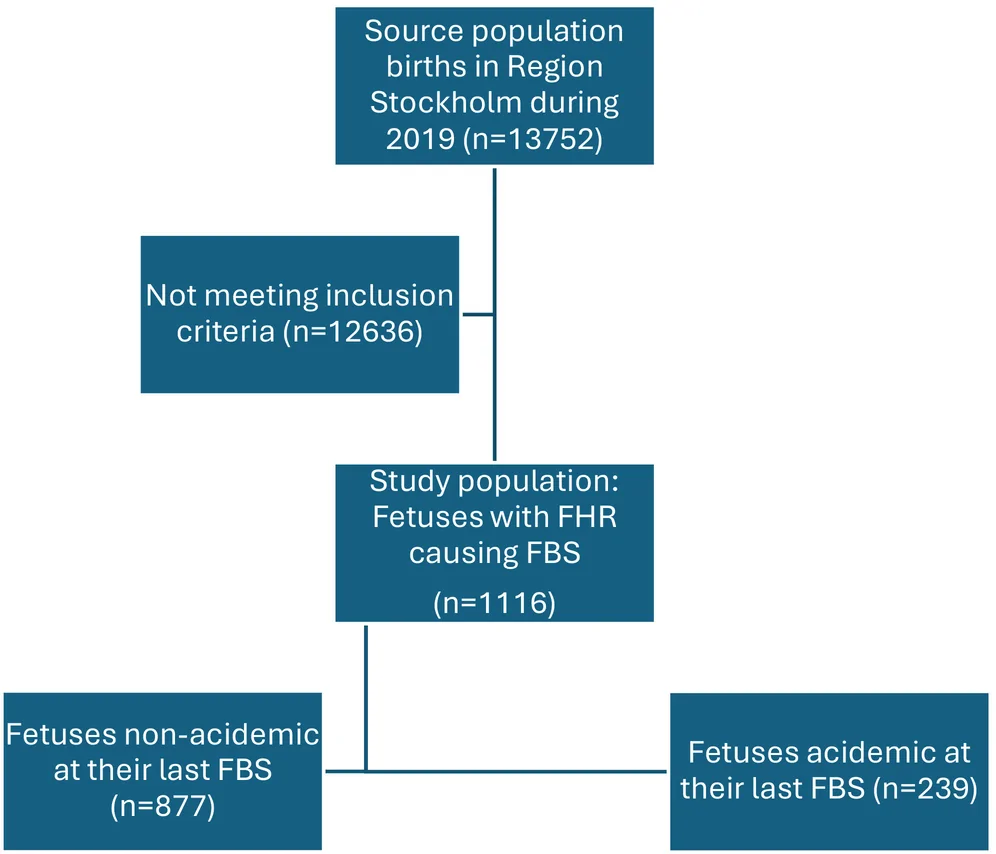
Flow chart of the study population.

In our data from the Stockholm‐Gotland Perinatal cohort, during the year 2019, there were a total of 13 752 deliveries. After inclusion criteria were used, 1116 mother‐fetus pairs (8.1%) were included, which entails all consecutive FBS's among the laboring population during the study period (Figure 1). Characteristics of the study population are shown in Table 1.

**TABLE 1:** Characteristics of study population (*n* = 1116), fetuses with and without acidemia at last fetal blood sampling, numbers (percentages), and medians (twenty‐fifth–seventy‐fifth percentile).

	Non‐acidemia (*n* = 877)	Acidemia^a^ (*n* = 239)
Maternal age (years)	31 (28–35)	33 (29–36)
Body mass index (kg/m^2^)	23.7 (21.2–27.1)	24.8 (22.1–27.8)
Gestational age (days)	284 (278–290)	285 (279–290)
Nulliparous *n* (%)	628 (71.6)	178 (74.5)
Use of oxytocin during labor *n* (%)	765 (87.2)	199 (83.3)
Delivery mode *n* (%)		
Spontaneous vaginal delivery	504 (57.5)	34 (14.2)
Instrumental vaginal delivery	199 (22.7)	106 (44.4)
Cesarean delivery	174 (19.8)	99 (41.4)
Birthweight (g)	3458 (3155–3780)	3456 (3180–3838)

^a^ Lactate concentration > 7.3 mmol/L with bedside meter Lactate Pro2.

For the 425 fetuses who underwent more than one FBS during labor, we present the first and the last FBS separately. Fetuses with only one FBS are included in both groups, that is, first and last sampling.

**FIGURE 2:**
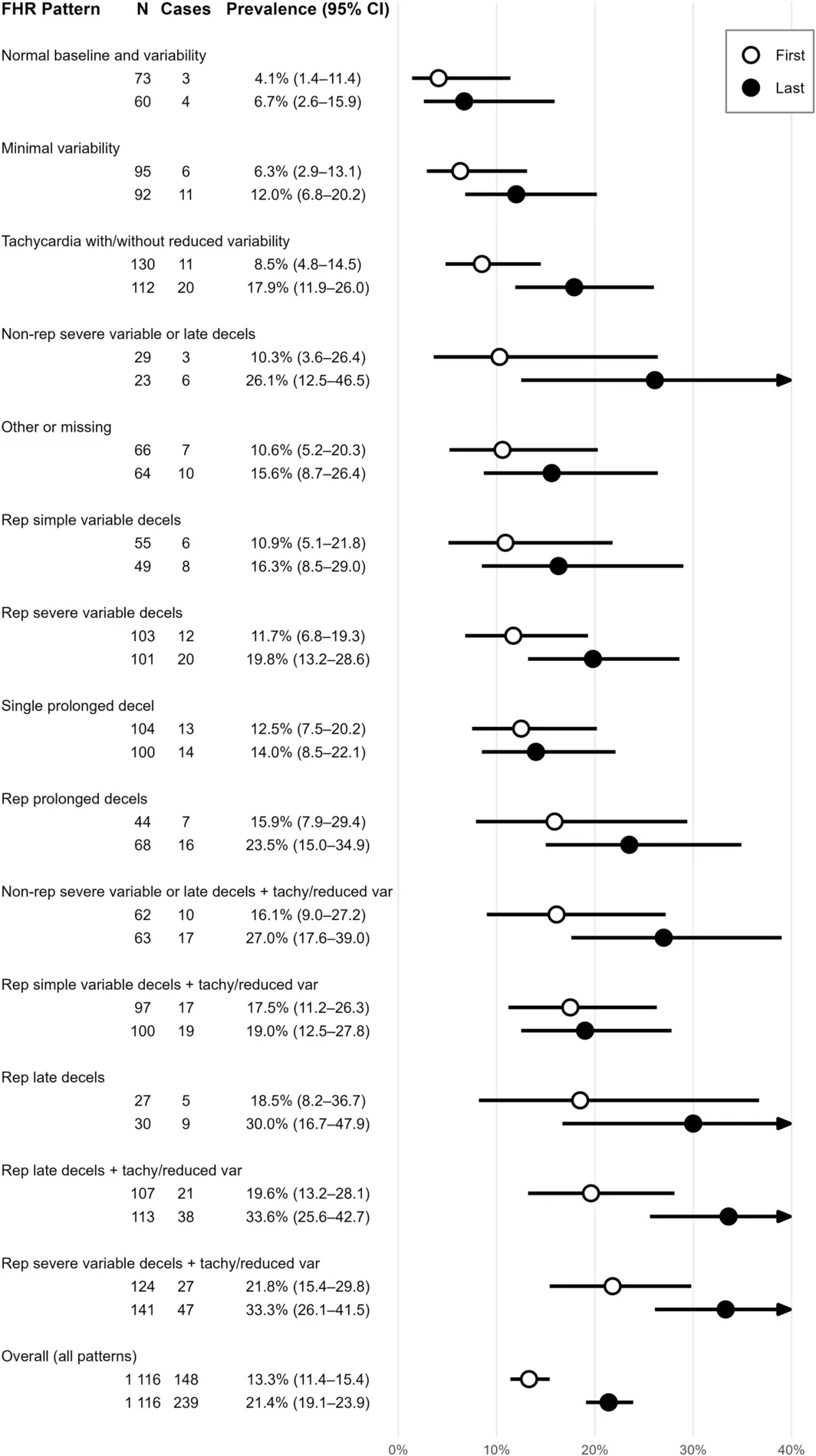
Numbers of FBS at various FHR abnormalities and frequencies of acidemia at first‐ and last‐ FBS. Decels = decelerations, non‐rep. = non‐repetitive, rep. = repetitive, tachy = tachycardia, var. = variability. Groups with absent variability, increased variability and bradycardia not calculated due to low numbers.

Different FHR patterns and their association with fetal acidemia are shown in Figure 2 and Tables 2 and 3. Figure 2 shows frequencies of acidemia with confidence intervals for different FHR patterns at the first‐ and last‐ FBS. Tables 2 and 3 present risk differences and odds ratios for fetal acidemia at the first last FBS, respectively.

**TABLE 2:** Fetal heart rate patterns, risk differences and odds ratios for fetal acidemia at the first FBS, Bonferroni‐adjusted *p*‐values.

FHR pattern	*N*	Cases^a^	RD vs. Ref (95% CI)	OR vs. Ref (95% CI)	Bonferroni‐ adjusted *p* ^b^
Normal baseline and variability^c^	73	3	0.0 (ref)	1.0 (ref)	NA
Minimal variability^d^	95	6	2.2 [−8.5 to 11.7]	1.57 [0.32–10.04]	1.000
Tachycardia ± reduced variability^e^	130	11	4.4 [−6.6 to 13.1]	2.16 [0.54–12.40]	1.000
Non‐repetitive severe variable + late decels^f^	29	3	6.2 [−7.8 to 25.0]	2.69 [0.34–21.17]	1.000
Non‐rep severe variable + late decels^f^ with tachycardia and/or reduced variability^e^	62	10	12.0 [−2.4 to 25.8]	4.49 [1.07–26.34]	0.280
Repetitive^g^ simple variable decels	55	6	6.8 [−6.3 to 20.4]	2.86 [0.57–18.35]	1.000
Repetitive^g^ simple variable decels with tachycardia and/or reduced variability^e^	97	17	13.4 [−0.2 to 24.9]	4.96 [1.34–27.28]	0.099
Repetitive^g^ severe variable decels	103	12	7.5 [−4.6 to 17.9]	3.08 [0.78–17.54]	1.000
Repetitive^g^ severe variable decels with tachycardia and/or reduced variability^e^	124	27	17.7 [4.0–28.4]	6.49 [1.87–34.49]	0.010
Repetitive^g^ late decels^f^	27	5	14.4 [−3.2 to 35.3]	5.30 [0.93–36.15]	0.410
Repetitive^g^ late decels^f^ with tachycardia and/or reduced variability^e^	107	21	15.5 [1.8–26.7]	5.70 [1.59–30.81]	0.039
Single prolonged deceleration	104	13	8.4 [−3.9 to 18.8]	3.33 [0.86–18.82]	0.847
Repetitive^g^ prolonged decels	44	7	11.8 [−3.5 to 28.0]	4.41 [0.93–27.61]	0.514
Other, undefined or missing CTG^h^	66	7	6.5 [−6.2 to 18.9]	2.77 [0.59–17.20]	1.000
Total	1116	148	–	–	–

*Note*: Decelerations specified as: simple variable (deceleration duration <1 min), severe variable (deceleration duration ≥1 min), prolonged (deceleration duration >3 min).

^a^ Lactate concentration > 7.3 mmol/L with bedside meter Lactate Pro2.

^b^ *p*‐values were adjusted for 13 comparisons with the Bonferroni method and adjusted *p*‐values <0.05 were considered statistically significant.

^c^ Including non‐repetitive early and simple variable decelerations.

^d^ Including reduced and absent variability, if absent, irrespective of other features.

^e^ Including variability 2–5 bpm.

^f^ Including combined decelerations.

^g^ Repetitive decelerations occur with ≥50% of contractions.

^h^ Including increased variability and bradycardia.

**TABLE 3:** Fetal heart rate patterns, risk differences and odds ratios for fetal acidemia at the last FBS, Bonferroni‐adjusted *p*‐values.

FHR pattern	*N*	Cases^a^	RD vs. Ref (95% CI)	OR vs. Ref (95% CI)	Bonferroni‐adjusted *p* ^b^
Normal baseline and variability^c^	60	4	0.0 (ref)	1.0 (ref)	NA
Minimal variability^d^	92	11	5.3 [−9.1 to 17.5]	1.90 [0.53–8.5]	1.000
Tachycardia ± reduced variability^e^	112	20	11.2 [−4.1 to 23.4]	3.04 [0.95–12.80]	0.818
Non‐repetitive severe variable + late decels^f^	23	6	19.4 [−3.4 to 43.8]	4.94 [1.01–26.11]	0.313
Non‐rep. severe variable + late decels^f^ with tachycardia and/or reduced variability^e^	63	17	20.3 [1.7 to 36.4]	5.17 [1.52–22.34]	0.046
Repetitive^g^ simple variable decels	49	8	9.7 [−7.4 to 26.4]	2.73 [0.67–13.14]	1.000
Repetitive^g^ simple variable decels with tachycardia and/or reduced variability^e^	100	19	12.3 [−3.4 to 25.2]	3.28 [1.01–13.90]	0.474
Repetitive^g^ severe variable decels	101	20	13.1 [−2.7 to 26.0]	3.46 [1.07–14.56]	0.310
Repetitive^g^ severe variable decels with tachycardia and/or reduced variability^e^	141	47	26.7 [10.2–38.8]	7.00 [2.35–27.96]	0.000
Repetitive^g^ late decels^f^	30	9	23.3 [0.7–45.3]	6.00 [1.45–28.95]	0.105
Repetitive^g^ late decels^f^ with tachycardia and/or reduced variability^e^	113	38	27.0 [9.7–40.1]	7.09 [2.33–28.70]	0.001
Single prolonged deceleration	100	14	7.3 [−7.4 to 19.5]	2.28 [0.67–9.95]	1.000
Repetitive^g^ prolonged decels	68	16	16.9 [−0.9 to 32.2]	4.31 [1.26–18.66]	0.173
Other, undefined or missing CTG^h^	64	10	9.0 [−7.2 to 23.8]	2.59 [0.69–11.93]	1.000
Total	1116	239	–	–	–

*Note*: Decelerations specified as: simple variable (deceleration duration <1 min), severe variable (deceleration duration ≥ 1 min), prolonged (deceleration duration > 3 min).

^a^ Lactate concentration > 7.3 mmol/L with bedside meter Lactate Pro2.

^b^ *p*‐values were adjusted for 13 comparisons with the Bonferroni method and adjusted *p*‐values < 0.05 were considered statistically significant.

^c^ Including non‐repetitive early and simple variable decelerations.

^d^ Including reduced and absent variability, if absent, irrespective of other features.

^e^ Including variability 2–5 bpm.

^f^ Including combined decelerations.

^g^ Repetitive decelerations occur with ≥50% of contractions.

^h^ Including increased variability and bradycardia.

At the first FBS, 73/1116 (6.5%) of the FHR patterns were retrospectively interpreted by the observer (MH) as normal according to the current Swedish classification. The corresponding numbers for the last FBS were 60/1116 (5.3%). Those fetuses were assessed as having a normal baseline and variability, and non‐repetitive early or simple variable decelerations could be present. These groups were used as the reference in the odds ratio calculations.

The most common FHR indication for FBS was tachycardia and/or decreased variability with repetitive severe variable decelerations, both at the first and last FBS (124/1116 [11%] and 141/1116 [12.7%] respectively). The second most common FHR pattern was tachycardia and/or reduced variability with repetitive late decelerations (107/1116 [9.6%] and 113/1116 [10.1%] respectively). Repetitive severe variable decelerations with normal baseline and variability as well as single prolonged decelerations were equally common reasons for FBS (100–104/1116 [≈9%], respectively) (Figure 2 and Tables 2 and 3).

The FHR patterns with the highest frequency of fetal acidemia were tachycardia and/or decreased variability with repetitive severe variable or late decelerations (21.8% and 19.6% at the first FBS, 33.3% and 33.6% at the last FBS, respectively).

At the last FBS, there were FHR patterns with potentially increased risk of fetal acidemia, but not classified as pathological in the revised Swedish CTG classification (except for patterns with reduced variability >60 min), that is,:

1. repetitive simple variable decelerations with tachycardia and/or reduced variability (19/100 with acidemia at the last FBS),
2. repetitive severe variable decelerations with normal baseline frequency and variability (20/101 with acidemia at the last FBS),
3. non‐repetitive severe variable or late with,

- normal baseline frequency and variability (6/23 with acidemia at the last FBS), and
- tachycardia and/or reduced variability (17/63 with acidemia at the last FBS).

**FIGURE 3:**
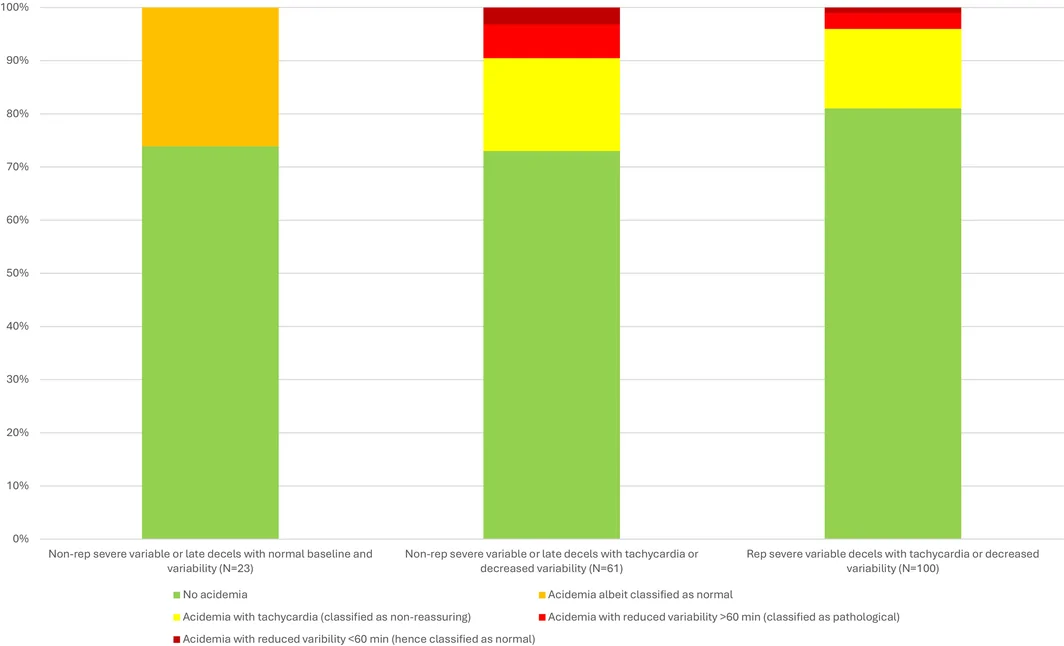
Fetal patterns potentially classified as normal in the revised classification, proportion of acidemia at the last FBS.

When performing a sub‐analysis of the data in Group 1, 15/19 fetuses with acidemia exhibited tachycardia, hence classified as non‐reassuring FHR. Three fetuses had decreased variability since >60 min that is, a pathological pattern. The entire group 2 is classified as non‐reassuring. As for group 3b, 11/17 of the fetuses had tachycardia. Four had reduced variability >60 min that is, a pathological pattern and less than three experienced a reduced variability <60 min, hence classified as normal. (Figure 3) The lactate concentrations in Group 3a ranged between 7.4–10.2 mmol/L with a median value of 7.8. All of the six fetuses had been exposed to the FHR pattern for >60 min.

In the analysis of the last FBS during labor, we lacked information about the time point of the beginning of pushing in 38.9% (*n* = 434) of the participants. Of those with known time of pushing, 162 had commenced pushing before the last FBS was collected. Of those, 19.8% (*n* = 32/162) had fetal acidemia at the last FBS.

## 4 DISCUSSION

Consistent with the previous study during the former CTG classification,^17^ we demonstrate that a FHR pattern with tachycardia and/or reduced variability in combination with repetitive either late decelerations or severe variable decelerations has the highest association with fetal acidemia at FBS. Among fetuses presenting with repetitive severe variable decelerations with tachycardia and/or reduced variability, 22% and 33% had increased lactate concentrations in their first and last FBS, respectively. Corresponding numbers for fetuses having a FHR with repetitive late decelerations, tachycardia and/or reduced variability were 20% and 34%, respectively.

All FHR patterns with the highest proportion of acidosis in our study are, according to the current Swedish CTG classification,^26^ interpreted as abnormal, except for one specific finding. In the last FBS, fetuses with non‐repetitive decelerations of any of the types severe variable decelerations, combined decelerations, or late decelerations in combination with a normal baseline frequency and normal variability showed approximately fivefold higher odds of acidosis, compared with the reference group. Indeed, 6/23 (26%) of the fetuses with this FHR pattern had acidemia at the last FBS and all of them were exposed to the specific FHR pattern for >60 min. With the revised Swedish classification, this specific pattern would be classified as normal. However, the numbers are small and must be interpreted with caution, due to the risk of a statistical chance finding with the use of multiple different CTG pattern groups.

Prior to the Bonferroni adjustment, a FHR pattern with repetitive severe variable decelerations with normal baseline and variability was associated with an increased proportion of acidemia at the last FBS, but the association was no longer statistically significant after Bonferroni adjustment. This pattern is considered non‐reassuring but not pathological with the revised classification. Twenty of 101 (20%) of the fetuses with this FHR pattern had increased lactate concentration at the last FBS, whereof 13 (65%) had been exposed to the abnormal CTG pattern >60 min.

Repetitive simple variable decelerations with tachycardia and/or reduced variability entailed an increased association with acidosis both in the first and last FBS in the initial analysis, 17/97 (18%) and 19/100 (19%) respectively, but the association was not statistically significant after Bonferroni adjustment. Simple variable decelerations are not considered abnormal in the revised Swedish CTG classification. However, reduced variability during >60 min is considered pathological and tachycardia >160 bpm as a non‐reassuring FHR pattern. As seen in Figure 3, the majority of the acidemic fetuses in this specific group exhibited tachycardia, that is, a non‐reassuring pattern. Only one fetus had a decreased variability less than 60 min, thereby classified as normal FHR pattern according to the revised Swedish classification.

To summarize the above, in our study, only 1% of all fetuses with repetitive simple variable decelerations with tachycardia and/or reduced variability would experience acidemia and be classified as a normal FHR with the revised Swedish CTG classification. More importantly, fetuses with non‐repetitive severe variable or late decelerations might have an increased risk of acidosis regardless of baseline frequency or variability. We also must consider the fact that some fetuses with these normal patterns according to the classification could have been withheld from FBS because of lack of indication. Therefore, the true proportion of acidemia with these FHR patterns is still unknown. A recent study has demonstrated an increased risk of low pH, low Apgar scores, and neonatal morbidity when comparing the period after the implementation of the revised CTG classification to the time before implementation. This risk of negative outcomes was increased both in the group of fetuses undergoing FBS as well as those who had not.^27^

We want to address the dilemma of discriminating between a non‐reassuring and a pathological FHR pattern. According to the revised Swedish CTG classification, a non‐reassuring pattern is associated with a low risk of fetal hypoxia and mandates continuing CTG registration, undertaking conservative measures for correction of possible underlying causes, and considering fetal scalp stimulation test or FBS. As for a pathological FHR pattern, the recommendation is to undertake conservative measures and perform scalp stimulation test or FBS or to expedite delivery.

We conclude that according to our study results the FHR patterns accompanied with the highest frequency of acidemia at last FBS are indeed categorized as pathological with a risk of >30% of acidemia at FBS. Conversely, several FHR patterns with a 19%–27% proportion of acidemia at the last FBS will merely be classified as non‐reassuring. It could be argued whether this proportion should be regarded as a “low risk of hypoxia” in the Swedish classification, or to be rephrased. However, at the time of the classification revision process, observational data revealed an acidemia frequency of 14% at FBS among fetuses with normal baseline and variability and severe variable decelerations.^17^ The cutoff for acidemia at FBS has been established to minimize the risk of a compromised neonate, that is, to prevent and not to predict neonatal acidemia in umbilical artery. According to our results from real‐world data with the use of the revised Swedish CTG classification and FBS, it is recommendable to rephrase the risk level and recommended measures for the non‐reassuring FHR patterns.

There has been a discussion in Sweden, whether the revised CTG classification has been too liberal in normalizing FHR patterns and subsequently has led to an increased risk for neonatal distress.^13^, ^14^, ^15^ In our study, the risk of fetal acidemia at FBS in the reference group was 4.1% at the first FBS and 6.7% at the last FBS. Corresponding numbers in the study from 2015 are 2.5% and 4.8%. However, the groups are not entirely comparable since the inclusion of decelerations in the reference group differs in the two studies; in the present study, early and non‐repetitive simple variable decelerations are included in the reference group and in the former study early, and non‐repetitive and repetitive simple variable decelerations. It is also important to note that, although retrospectively classified as the reference group, the assessment was blinded to maternal risk factors, stage of labor etc., and in the clinical situation, FBS was performed upon indication in those cases according to the doctor on call. Moreover, the limit of tachycardia differs with a present cutoff of 160 bpm compared to the previous 150 bpm limit. Meanwhile, the incidence of FBS has decreased since the previous study.^27^

Most commonly, women in the active phase of the second stage of labor have been excluded from FBS studies due to the shown increased risk of elevated lactate concentration after start of pushing^25^ In our study, we did not exclude women in the active second stage of labor due to a high proportion of missing data regarding the beginning of the active phase of the second stage. Among those with information on commenced pushing in the last FBS group, >80% of fetuses had a normal lactate concentration. This might suggest that FBS does not need to be discouraged during pushing as one of the factors to assess fetal status. However, the numbers should be interpreted with caution since FBS in the active stage of labor was not the scope of our study. If used, it should be performed with cautious consideration that fetal acidemia might develop faster during the active second stage of labor than during earlier stages of labor.^28^, ^29^

The use of FBS varies between countries partly due to the invasiveness and potential risks for complications, mainly bleeding and infection. With modern equipment, the risks are said to be no greater than the use of scalp electrodes for internal monitoring of the FHR. Schaap et al. described the risks for severe complications with FBS as low as 0.08%^30^ and the risk assessment included case reports with the use of scalpels not in clinical use since many years. More recent studies showed neonatal complication frequencies after fetal scalp electrode not higher than instrumental vaginal birth, which in many clinical situations would be the alternative intervention.^31^, ^32^

The benefit of FBS as an adjunct to CTG is debated with limited evidence from observational studies and beyond the scope of this study. However, considering the results in our cohort, the numbers needed to detect (NND) one acidemic fetus would range from for example, NND 3 for tachycardic fetuses with normal or reduced variability and repetitive either late decelerations or severe variable decelerations and up to NND 5 for fetuses with normal baseline and variability but repetitive severe variable decelerations.

The use of increased lactate concentration at FBS as outcome and a proxy for fetal acidemia needs to be considered when assessing our results. Contamination with amniotic fluid can affect the lactate concentration and introduce misclassification bias. The risk is however low, considering the small amount of blood needed.^8^, ^22^ Fetal acidemia should not be confused with neonatal acidemia, since the cutoff for definition of fetal acidemia at FBS (with pH as well as lactate analysis) and recommendations for action are set to prevent acidemia in umbilical artery at birth if guidelines are followed.^29^ Increased lactate concentration in peripheral circulation such as fetal scalp capillary blood is also not fully equal to acidemia in central circulation and vital organs.^33^ However, large real‐live cohorts have shown a low risk for false negativity^9^ and carefully tuned balances between sensitivity and specificity.^29^, ^34^

Considering selection bias, we are aware that some abnormal FHR patterns, for example, bradycardia, prolonged decelerations or absent variability, in many cases indicate and lead to prompt delivery without prior FBS. These groups were too small to report separate results in our study.

In cases with a mix of simple and severe variable decelerations, the observer assessed the FHR pattern as repetitive severe variable decelerations, even when the severe decelerations occurred <50% of contractions. This is not strictly defined in the Swedish CTG classification and could therefore introduce misclassification bias.

Another limitation is that the group with a FHR pattern used as reference in our study, for some reason, had FBS performed due to the doctor on call interpreting the CTG as non‐reassuring or abnormal. There might also have been aspects of the delivery process, unknown to us but clinically considered abnormal, that led to FBS. FHR interpretation in the labor setting is and must always be integrated with clinical information for comprehensive interpretation and adequate management.^10^

Despite a similar total sample size as the previous study with corresponding study design,^17^ the reference group in the current study was much smaller, entailing a larger uncertainty of our results. We could not extend the study period further due to the latest update of the research cohort used in this study.^18^ The size difference between the reference groups in the two cohorts is probably due to the former CTG classification being stricter, which gave rise to more liberal use of FBS on milder FHR alterations such as lack of accelerations. Due to many groupwise comparisons, our results need to be interpreted with caution and as hypothesis‐generating, especially regarding the smaller subgroups, and groups with lower frequency of acidemia.

Our material solely consists of fetuses undergoing FBS on indication, which could introduce selection bias. If a non‐selected population would be possible to measure, we could speculate that the risk of acidosis in the reference group would decrease, and hence the estimates of the odds ratios comparing the different FHR patterns to the reference group might be underestimated.

Another limitation is the CTG interpretation of one single observer. We aimed to minimize the hindsight bias and intra‐observer variation by blinding the FBS results and having a short interpretation period. The CTG interpreter has in a previous study shown moderate to substantial interobserver reliability and substantial to excellent intra‐observer reliability.^35^ Previous studies have shown good agreement when interpreting FHR patterns that occur more frequently. FHR alterations that occur more seldom, for example, saltatory patterns, show a larger interobserver difference.^36^ Interobserver agreement between senior and junior doctors is also higher on normal FHR patterns compared to abnormal or pathological patterns.^37^

## 5 CONCLUSION

In a high‐risk cohort of fetuses with FBS upon clinical indication, all FHR patterns with the highest risk of acidemia are considered abnormal according to the revised Swedish CTG classification. The risk for fetuses with long‐standing non‐repetitive severe variable or late decelerations needs further studies. We conclude that the FHR patterns most often associated with fetal acidemia during labor are tachycardia and/or reduced variability combined with repetitive late or severe variable decelerations.

## AUTHOR CONTRIBUTIONS

MH initiated the study concept. MH, KG, EG, and DM contributed to the study design. KJ was responsible for the data collection from the register. MH and KG collected the CTG data, and MH interpreted the CTGs. DM and EG performed the analysis of the data. DM wrote the first draft of the manuscript. All authors contributed with their intellectual property in the manuscript and revised the manuscript.

## FUNDING INFORMATION

Malin Holzmann was supported by Region Stockholm (clinical research appointment). Erika Gyllencreutz was supported by the Unit of Research, Education and Development, Region Jämtland Härjedalen.

## CONFLICT OF INTEREST STATEMENT

The authors have no conflicts of interest to declare.

## ETHICS STATEMENT

Ethical approval was granted by the Swedish Ethical Review Authority on April 2, 2009 (2009/275‐31) and July 3, 2019 (2019‐02818). The requirement for consent was waived by the review authority due to the historical study design and results presented in aggregated form. The data included in this study were based on Swedish electronic medical records and national health‐care registers and cannot be made publicly available. According to Swedish law, data sharing is not allowed, as individual‐level data in the medical records and registers can only be accessed through secure servers, and only the export of aggregated data is permitted. Data can only be shared for specific collaboration projects between Karolinska Institutet as the host and the collaborating university by signing data process agreements and a research collaboration agreement.

## Data Availability

Research data are not shared.

## References

[1] Alfirevic Z , Devane D , Gyte GM , Cuthbert A . Continuous cardiotocography (CTG) as a form of electronic fetal monitoring (EFM) for fetal assessment during labour. Cochrane Database Syst Rev. 2017;2(2):Cd006066. 28157275 10.1002/14651858.CD006066.pub3 PMC6464257

[2] Hernandez Engelhart C , Gundro Brurberg K , Aanstad KJ , et al. Reliability and agreement in intrapartum fetal heart rate monitoring interpretation: a systematic review. Acta Obstet Gynecol Scand. 2023;102(8):970‐985. 37310765 10.1111/aogs.14591 PMC10378030

[3] Jørgensen JS , Weber T . Fetal scalp blood sampling in labor‐‐a review. Acta Obstet Gynecol Scand. 2014;93(6):548‐555. 24806978 10.1111/aogs.12421

[4] Reif P , Haas J , Schöll W , Lang U . Foetal scalp blood sampling: impact on the incidence of caesarean section and assisted vaginal deliveries for non‐reassuring foetal heart rate and its use according to gestational age. Z Geburtshilfe Neonatol. 2011;215(5):194‐198. 22028059 10.1055/s-0031-1287861

[5] Bretscher J , Saling E . pH values in the human fetus during labor. Am J Obstet Gynecol. 1967;97(7):906‐911. 6021106 10.1016/0002-9378(67)90515-7

[6] Nordström L , Ingemarsson I , Kublickas M , Persson B , Shimojo N , Westgren M . Scalp blood lactate: a new test strip method for monitoring fetal wellbeing in labour. Br J Obstet Gynaecol. 1995;102(11):894‐899. 8534626 10.1111/j.1471-0528.1995.tb10878.x

[7] East CE , Leader LR , Sheehan P , Henshall NE , Colditz PB , Lau R . Intrapartum fetal scalp lactate sampling for fetal assessment in the presence of a non‐reassuring fetal heart rate trace. Cochrane Database Syst Rev. 2015;2015(5):Cd006174. 25929461 10.1002/14651858.CD006174.pub3 PMC10823414

[8] Westgren M , Kruger K , Ek S , et al. Lactate compared with pH analysis at fetal scalp blood sampling: a prospective randomised study. Br J Obstet Gynaecol. 1998;105(1):29‐33. 9442158 10.1111/j.1471-0528.1998.tb09346.x

[9] Wiberg‐Itzel E , Lipponer C , Norman M , et al. Determination of pH or lactate in fetal scalp blood in management of intrapartum fetal distress: randomised controlled multicentre trial. BMJ. 2008;336(7656):1284‐1287. 18503103 10.1136/bmj.39553.406991.25 PMC2413392

[10] Ayres‐de‐Campos D , Spong CY , Chandraharan E . FIGO consensus guidelines on intrapartum fetal monitoring: cardiotocography. Int J Gynaecol Obstet. 2015;131(1):13‐24. 26433401 10.1016/j.ijgo.2015.06.020

[11] Holzmann M , Jonsson M , Nilsson M , Herbst A , Ladfors L , Nordström L . Nya svenska riktlinjer för CTG‐tolkning under förlossning. Medlemsbl. 2016;4:33‐34.

[12] group TSCcw . Education and training in CTG interpretation. Intrapartalt CTG www.ctgutbildning.se: The Swedish Patient Insurance Company; 2019. http://ctgutbildning.se/index.php/utbildningskapitel/klassificering‐av‐ctg/intrapartalt‐ctg

[13] Jonsson M , Söderling J , Ladfors L , et al. Implementation of a revised classification for intrapartum fetal heart rate monitoring and association to birth outcome: a national cohort study. Acta Obstet Gynecol Scand. 2022;101(2):183‐192. 35092004 10.1111/aogs.14296 PMC9564816

[14] Ekengård F , Cardell M , Herbst A . Impaired validity of the new FIGO and Swedish CTG classification templates to identify fetal acidosis in the first stage of labor. J Matern Fetal Neonatal Med. 2022;35(25):4853‐4860. 33406946 10.1080/14767058.2020.1869931

[15] Ekengård F , Cardell M , Herbst A . Low sensitivity of the new FIGO classification system for electronic fetal monitoring to identify fetal acidosis in the second stage of labor. Eur J Obstet Gynecol Reprod Biol X. 2021;9:100120. 33319210 10.1016/j.eurox.2020.100120 PMC7724159

[16] Olofsson P , Ekengård F , Herbst A . Time to reconsider: have the 2015 FIGO and 2017 Swedish intrapartum cardiotocogram classifications led us from Charybdis to Scylla? Acta Obstet Gynecol Scand. 2021;100(9):1549‐1556. 34060661 10.1111/aogs.14201

[17] Holzmann M , Wretler S , Cnattingius S , Nordström L . Cardiotocography patterns and risk of intrapartum fetal acidemia. J Perinat Med. 2015;43(4):473‐479. 24914710 10.1515/jpm-2014-0105

[18] Johansson K , Granfors M , Petersson G , et al. The Stockholm‐Gotland perinatal cohort‐a population‐based cohort including longitudinal data throughout pregnancy and the postpartum period. Paediatr Perinat Epidemiol. 2023;37(4):276‐286. 36560891 10.1111/ppe.12945

[19] Jonsson M , Holzmann M , Rilby L , Elvander C . Fosterövervakning i samband med förlossning. National recommendations for clinical guidelines. 2022. Accessed June 15, 2022. https://lof.se/patientsakerhet/vara‐projekt/saker‐forlossningsvard/rekommendationer‐och‐rad

[20] Birgisdottir BT , Holzmann M , Varli IH , Graner S , Saltvedt S , Nordström L . Reference values for lactate pro 2™ in fetal blood sampling during labor: a cross‐sectional study. J Perinat Med. 2017;45(3):321‐325. 27089399 10.1515/jpm-2016-0027

[21] Iorizzo L , Klausen TW , Wiberg‐Itzel E , Ovin F , Wiberg N . Use of lactate pro(TM)2 for measurement of fetal scalp blood lactate during labor ‐ proposing new cutoffs for normality, preacidemia and acidemia: a cross‐sectional study. J Matern Fetal Neonatal Med. 2019;32(11):1762‐1768. 29301439 10.1080/14767058.2017.1416603

[22] Wiberg‐Itzel E . Amniotic fluid lactate (AFL): a new predictor of labor outcome in dystocic deliveries. J Matern Fetal Neonatal Med. 2022;35(25):7306‐7311. 34758684 10.1080/14767058.2021.1946790

[23] Wilson EB . Probable inference, the law of succession, and statistical inference. J Am Stat Assoc. 1927;22(158):209‐212.

[24] Newcombe RG . Interval estimation for the difference between independent proportions: comparison of eleven methods. Stat Med. 1998;17(8):873‐890. 9595617 10.1002/(sici)1097-0258(19980430)17:8<873::aid-sim779>3.0.co;2-i

[25] Fisher RA . On the interpretation of χ^2^ from contingency tables, and the calculation of P. J R Stat Soc Ser A (Stat Soc). 1922;85(1):87‐94.

[26] SFOG Ssfoag, SBF SAfm . Svenska riktlinjer för bedömning av intrapartalt CTG. www.ctgutbildning.se. www.lof.se/patientsakerhet/vara‐projekt/saker‐forlossningsvard/rekommendationer‐och‐rad2019; https://wp.lof.se/wp‐content/uploads/INTRA‐partalt‐ctg_tryck.pdf

[27] Gröndal K , Gyllencreutz E , Wretler S , Johansson K , Holzmann M . Frequency of fetal blood sampling, delivery mode and neonatal outcome after revised CTG‐classification and updated lactate meter in Sweden: an observational study. Acta Obstet Gynecol Scand. 2025;104(4):676‐684. 39917811 10.1111/aogs.15063 PMC11919722

[28] Nordström L , Achanna S , Naka K , Arulkumaran S . Fetal and maternal lactate increase during active second stage of labour. BJOG. 2001;108(3):263‐268. 11281466 10.1111/j.1471-0528.2001.00034.x

[29] Iorizzo L , Carlsson Y , Johansson C , et al. Proposed cutoff for fetal scalp blood lactate in intrapartum fetal surveillance based on neonatal outcomes: a large prospective observational study. BJOG. 2022;129(4):636‐646. 34555249 10.1111/1471-0528.16924

[30] Schaap TP , Moormann KA , Becker JH , et al. Cerebrospinal fluid leakage, an uncommon complication of fetal blood sampling: a case report and review of the literature. Obstet Gynecol Surv. 2011;66(1):42‐46. 21510911 10.1097/OGX.0b013e318213e644

[31] Kawakita T , Reddy UM , Landy HJ , Iqbal SN , Huang CC , Grantz KL . Neonatal complications associated with use of fetal scalp electrode: a retrospective study. BJOG. 2016;123(11):1797‐1803. 26643181 10.1111/1471-0528.13817 PMC4899296

[32] Fick T , Woerdeman PA . Neonatal brain abscess development following fetal scalp electrode placement: a rare complication. Childs Nerv Syst. 2022;38(1):199‐202. doi:10.1007/s00381-021-05150-7 33825051 PMC8724088

[33] Kastendieck E , Paulick R , Martius J . Lactate in fetal tissue during hypoxia; correlation to lactate, pH and base deficit in the fetal blood. Eur J Obstet Gynecol Reprod Biol. 1988;29(1):61‐71. 3224744 10.1016/0028-2243(88)90166-9

[34] Kruger K , Hallberg B , Blennow M , Kublickas M , Westgren M . Predictive value of fetal scalp blood lactate concentration and pH as markers of neurologic disability. Am J Obstet Gynecol. 1999;181(5 Pt 1):1072‐1078. doi:10.1016/s0002-9378(99)70083-9 10561620

[35] Gyllencreutz E , Hulthén Varli I , Lindqvist PG , Holzmann M . Reliability in cardiotocography interpretation—impact of extended on‐site education in addition to web‐based learning: an observational study. Acta Obstet Gynecol Scand. 2017;96(4):496‐502. 28052320 10.1111/aogs.13090

[36] Rei M , Tavares S , Pinto P , et al. Interobserver agreement in CTG interpretation using the 2015 FIGO guidelines for intrapartum fetal monitoring. Eur J Obstet Gynecol Reprod Biol. 2016;205:27‐31. 27566218 10.1016/j.ejogrb.2016.08.017

[37] Palomäki O , Luukkaala T , Luoto R , Tuimala R . Intrapartum cardiotocography—the dilemma of interpretational variation. J Perinat Med. 2006;34(4):298‐302. 16856819 10.1515/JPM.2006.057

